# The Paternal Aspect and Relation of the United States Government to Science

**Published:** 1906-07

**Authors:** 


### The Paternal Aspect and Relation of the United States Government to S.ience.

The *Medico-Legal Journal,* commenting on the letters of Secretary of State Root and Assistant Secretary Bacon (reproduced in this issue), and publishing them in full, says editorially:

"The American Government has taken strong ground in aid of preventive legislation in the conflict with tuberculosis.

"The American International Congress on Tuberculosis has decided to make a renewal of the assault along the same lines, on which it won its victories at the St. Louis Congress, held at the World's Exposition, in October, 1904.

"Hon. Elihu Root brings the splendid sympathetic power of the great republic of the world's civilization in support of the aims and purposes of the American International Congress on Tuberculosis, to be held in the American metropolis, November 14, 15, and 16, 1906.

"He shows great foresight, wisdom and statesmanship in placing the whole moral force of our government behind the great purpose of the American International Congress on Tuberculosis.

"He has used the same splendidly sympathetic language in bringing it to the official notice of all the governments in the western hemisphere, through our Diplomatic corps, that Mr. Secretary Hay employed in recommending the St. Louis Congress to foreign governments.

"Mr. Root is making history for both our government and for our people. The language employed is worthy of the cause, worthy of the occasion.

"The battle cry of the Congress is Preventive Legislation Against Tuberculosis—To arrest, to avert, to minimize the spread of Consumption, is the battle ground.

"The call of the Congress is to the masses of the people, to the men of all professions, the statesman, the publicist, the humanitarian.

"It is not a medical question; not confined to medical men, but the call is to all men of all professions, and to the gigantic proportions of the conflict, and the magnitude of the problems, which now confront the health and the safety of that great, that enormous mass of human lives that have been yearly sacrificed to the ravages of this dreadful disease."

